# Prevalence of human papillomavirus infection and genotype distribution among high-risk Korean women for prospecting the strategy of vaccine development

**DOI:** 10.1186/1743-422X-7-201

**Published:** 2010-08-25

**Authors:** Jee Eun Rhee, Mi Yeong Shin, Choong Mo Kim, Hye Young Kee, Jae Keun Chung, Sang-Kee Min, Seong-Joon Kim, Dai-Ho Jang, Sung Soon Kim, Byeong-Sun Choi

**Affiliations:** 1 Division of AIDS, Center for Immunology and Pathology, National Institute of Health, Korea Centers for Disease Control and Prevention, Seoul, Korea; 2 Division of Microbiology, Jeollanam-Do Institute of Health and Environment, Jeollanamdo, Korea; 3 Department of Health Research, Gwangju Institute of Health and Environment, Gwangju, Korea; 4 Division of Epidemiology, Busan Institute of Health and Environment, Busan, Korea

## Abstract

We investigated the prevalence of human papillomavirus (HPV) infection and the distribution of high-risk HPV genotypes among 2,308 high-risk Korean women to predict how much the current prophylactic HPV vaccines might affect the prevention of cervical cancer in Korea. HPV DNA was detected in 939 women (40.7%) but only one-third of women were positive for HPV-16 and/or HPV-18, the genotypes used for developing the HPV vaccines. Thus, the development of area-specific HPV vaccines based on dominant HPV genotypes in our country is needed for preventing HPV infection and the development of premalignant lesions in the cervix of Korean women.

## Findings

Infection with certain types of human papillomavirus (HPV) is recognized as a causal and necessary factor for developing cervical cancer [1,2], which is the fourth of the most common cancer in South Korean women [3]. More than 140 different HPV genotypes have been characterized and approximately 50 of these genotypes are known to infect the genital tract and be oncogenic or high-risk (HR) types (HPV-16, -18, -31, -33, -35, -39, -45, -51, -52, -56, -58, -59, -66 and -68). HR genotypes are significantly associated with progression to invasive cervical cancer [2,4]. Therefore, assessment of the HPV genotypic spectrum among sexually active women is important for predicting public health problems such as the risks of developing cervical intraepithelial neoplasia and cervical cancer [5,6].

HPV-16, the most common HR type, is detected in 50 - 60% of high-grade squamous intraepithelial lesions and invasive cervical cancers and HPV-18 is followed by an incidence of 10 - 20% [4,7]. Thus, HPV-16 and -18 are considered as the types responsible for causing most cervical cancers in many countries [4,7]. Clinical trials have reported that these vaccines can protect many uninfected women from developing precancerous cervical lesions caused by HPV-16 and -18 [8,9]. In spite of their high effectiveness to reduce the incidence of cervical cancer, the usefulness of these vaccines is still being debated because of the differences in the geographical distribution of HPV genotypes [10].

Vaccines against HPV-16 and -18 have been developed to help the prevention of cervical cancer and the use of Merck's Gardasil (the quadrivalent vaccine for HPV-6, -11, -16 and -18) and GSK's Cervarix (the bivalent vaccine for HPV-16 and -18) have been licensed by the Korea Food and Drug Administration. Population-based study for the distribution of HPV genotype is needed to predict how much these vaccines might influence to the prevention of cervical cancer.

In the present study, the prevalence and distribution of HPV genotypes among high-risk women, which are called as commercial sex workers (CSWs) by other countries, were examined to predict whether the developed HPV vaccines are sufficient for preventing HPV infection and the development of premalignant lesions of the cervix in South Korea.

A cohort of 2,308 high-risk women visiting for regular sexually transmitted infection testing in public health centers in four different regions (Seoul, Busan, Gwangju and Jeollanamdo) was enrolled in this study. Specimens were collected with a cytobrush for HPV testing, placed in viral transport medium (Cellmatics Viral Transport Pack, BD Diagnostics, Franklin Lakes, NJ, USA) and stored at 4°C until the use for experiment. Genomic DNA was extracted from cervical swabs by using an AccuPrep Genomic DNA Extraction Kit (Bioneer Co., Seoul, South Korea) according to the manufacturer's instructions. Purified DNA was used to detect HPV DNA and to determine the genotypes by using a HPV DNA Chip (Biocore Co. Ltd., Seoul, South Korea). 939 out of 2,308 specimens were HPV positive and HPV genotypes were typed using the HPV DNA Chip with 32 type-specific probes (HR types: HPV-16, -18, -26, -31, -33, -35, -39, -45, -51, -52, -53, -56, -58, -59, -66, -68 and -69; low-risk [LR] types: HPV-6, -11, -32, -34, -40, -42, -43, -44, -54, -55, -57, -61, -62, -70 and -73).

**Figure 1:**
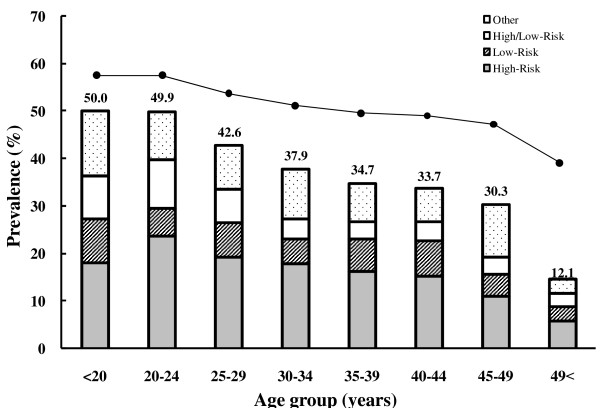
**The distribution of HPV prevalence by age group**. Age groups were defined based on age at enrollment into the study.

**Figure 2:**
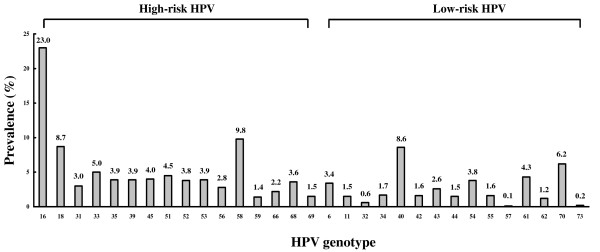
**Cross-sectional overview of HPV genotypic distribution among high-risk women in South Korea**. The total number of HPV-infected cases in each genotype, including single and multi-infection, was calculated as the percentage of the total number of infectious cases (n = 939).

The overall prevalence of HPV infection in these high-risk women was 40.7%. HPV genotypes among 939 HPV-infected women were detected in 431 (45.9%) for HR types and 147 (15.7%) for LR types, respectively. 147 (15.7%) women were infected with both genotypes. HPV prevalence was decreased with age and the highest HPV prevalence was observed in women under the age of 24 years old (Figure 1). The predominant HPV genotypes in this study were HPV-16 (23.0%), HPV-58 (9.8%), and HPV-18 (8.7%) (Figure 2). The number of HPV-infected women with HPV-16 and -18 were 107 (11.4%) and 43 (4.6%) in a single-type infection and 109 (11.6%) and 39 (4.2%) in multiple-type infections, respectively (Table 1).

**Table 1:** Distribution of high-risk women infected with HPV-16 and/or HPV-18 genotypes and with single or multiple infections by age group (n = 939)

Age (years)	No. of HPV-16-infected women (%)		No. of HPV-18-infected women (%)	
	
	Single	Multiple	Single	Multiple
< 20	1 (0.9)	3 (2.8)	0 (0.0)	2 (5.1)
20-24	28 (26.2)	17 (15.6)	11 (25.6)	6 (15.4)
25-29	31 (29.0)	41 (37.6)	17 (39.5)	12 (30.8)
30-34	22 (20.6)	7 (6.4)	7 (16.3)	3 (7.7)
35-39	13 (12.1)	4 (3.7)	5 (11.6)	0 (0.0)
40-44	6 (5.6)	5 (4.6)	4 (9.3)	2 (5.1)
45-49	5 (4.7)	1 (0.9)	0 (0.0)	0 (0.0)
> 49	1 (0.9)	0 (0.0)	0 (0.0)	0 (0.0)

Total	107 (11.4)	109 (11.6)	43 (4.6)	39 (4.1)

The prevalence of HPV infection in female CSWs from other countries was 57% in the Philippines [11], 39% in Spain [12], 32% in Australia [13] and 48% in Japan [14]. In our previous data [15], the prevalence of HPV infection in high-risk women was 47% with the Hybrid Capture II method (Digene Diagnostics). Although the HPV prevalence among CSWs differs between countries, it was much higher than among low-risk women and men [16]. In addition, the prevalence of HPV infection in this study was lower than our previous study. This result may be generated by higher sensitivity of the Hybrid Capture assay compared to the DNA chip method and different population examined. The higher prevalence of HPV infection in younger women and the decreasing trend with age has been described by many other studies [12,17]. The inverse relation between age and HPV prevalence has been attributed to the development of acquired HPV immunity over time after HPV exposure [17,18].

In this study, HPV-16 and -18 were detected only 31.7% including single or multiple infections in HPV-infected high-risk women (Table 1). The most prevalent HPV genotype was HPV-16 and next dominant type was the HPV-58 (Figure 2). According to Shin *et al*. [16], the prevalence of HPV infection for female university students in Busan was 15.2% and the common HPV genotypes were HPV-51, -53, -56, -16 and -52. In CSWs in the Philippines, HPV-52 was the most prevalent genotype and other dominant types were HPV-66, -16, -45 and -67 [11]. Among general gynecology practices in South Taiwan, the prevalent genotypes were HPV-16, -52, -58, -18 and -51 [19]. These results suggest that the predominant HPV genotypes such as HPV-58, -52 and -51 in Asian countries as well as HPV-16 might play some important roles in cervical carcinogenesis in these countries.

Even if clinical trials of prophylactic vaccines targeted for HPV-16 and -18 showed dramatically preventive effect for HPV infection and precancerous lesions, the cross-protection between various HPV genotypes is still unsolved in vaccinated women [20]. These results indicate that area-specific HPV vaccines should be developed for preventing HPV infection and the subsequent development of premalignant lesions of the cervix in Korean women.

## List of abbreviations

HPV: human papillomavirus; HR: high-risk; LR: low-risk; CSWs: commercial sex workers

## Competing interests

The authors declare that they have no competing interests.

## Authors' contributions

MY, CM, HY, JK, SK, SJ collected the samples and carried out the experiments. DH and SS participated in the design of the study and supported performing of experiments. JE and BS designed the research and wrote and edited the manuscript. All authors read and approved the final manuscript.
